# Supplementary material for: A multi-strain, biofilm-forming cocktail of Bacillus spp. and Pediococcus spp. alters the microbial composition on polyethylene calf housing surfaces
Source: Microbiol Spectr. 2025 May 28;13(7):e03302-24. doi: 10.1128/spectrum.03302-24 (PMC12210986; doi:10.1128/spectrum.03302-24)
Supplement: Supplemental material — Supplemental figure and table. [file spectrum.03302-24-s0001.pdf]

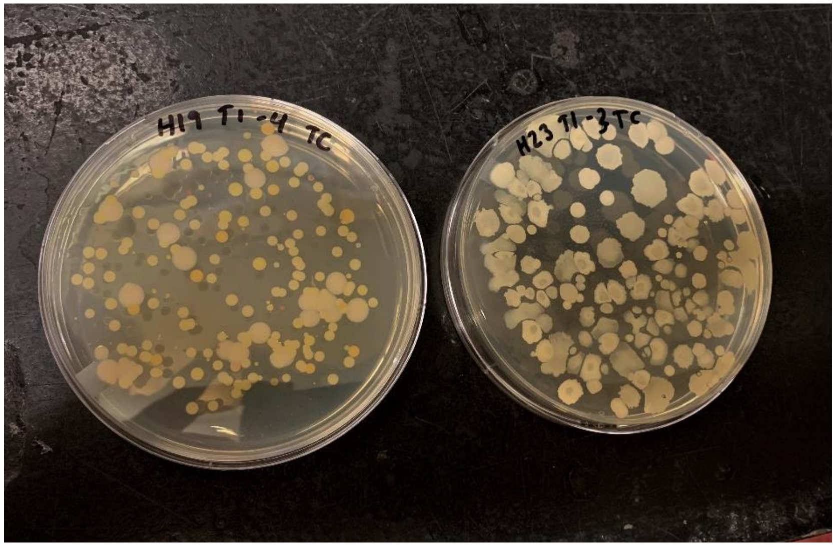

Supplementary Figure 1. Total cell counts on tryptic soy agar enumerated from samples obtained 24 h post-product application from polyethylene calf hutches treated with either a negative control (NC: no application) or a beneficial microbial cocktail (LF; Lallemand SAS, Blagnac, France) at a concentration of 0.4 g/m^2.

Supplementary Table 1. Parameters of RT-qPCR analysis performed to detect microbial DNA in situ over a 21-d treatment period[1] on polyethylene calf hutch surfaces treated with treated with a negative control (NC; no application), a positive control of distilled water (DW), or a beneficial microbial cocktail containing *Bacillus spp.* and *Pediococcus spp*. at a concentration of 0.4 g/m^2 (LF).

	Target			
Parameter	*Escherichia* spp.[2]	*Salmonella* spp.[3]	*Cryptosporidium parvum*[4]	Lactic Acid Bacteria[5]
Base pair amplicon sequence, 5’-3’	**F**:CGGAAGCAAC GCGTAAACTC **R**:TGATGGTATC GGTGTGAGCG	**F**:TCGTCATTCCATTA CCTACC **R**:AAACGTTGAAAAA CTGAGGA	**F**:CAAATTGATACCGTTTG TCCTTCTG **R**:GGCATGTCGATTCTAAT TCAGCT	**F:**AGCAGTAGGGAATCT TCCA **R**:CGCCACTGGTGTTCYT CCATATA
Primer concentration, µM	1.2	0.5	0.6	0.6
Amplification	95°C/3 min	95°C/1 min	95°C/10 min	95°C/3 min
Number of cycles	45	35	40	35
Denaturation	95°C/3 s	95°C/15 s	95°C/15 s	95°C/15 s
Annealing	60°C/10 s	52°C/30 s	60°C/1 min	60°C/1 min
Extension		72°C/35 s + 72°C/5 min		72°C/30 s
Denaturation				95°C/15 s
Melting curve	65-95°C (0.5°C/5 s)	60-90°C (0.5°C/5 s)	65-95°C (0.5°C/5 s)	65-95°C (0.5°C/5 s)
Positive Control	DNA from fecal sample of calf with *E. coli*	Fecal supernatant inoculated with *Salmonella typhimurium* DNA (ATCC 14028)	Calf fecal material inoculated with genomic DNA (ATCC PRA 67DQ)	DNA from *Lentilactobacillus buchneri* (CNCM 40788)
Standard Curve	DNA from fecal isolate of *E. coli*	DNA extracted from *Salmonella typhimurium* (ATCC 14028)	Genomic DNA (ATCC PRA 67DQ)	DNA from *Lactiplantibacillus plantarum* NCIMB 12422

[1]Treatments were a negative control (NC: no application), a positive control of distilled water (DW) or a beneficial microbial cocktail (LF; Lallemand SAS, Blagnac, France) at a concentration of 0.4 g/m^2. [2]Miotto et al., 2019 [3]Nurjanah et al., 2018 [4]Guy et al., 2003 [5]Furet et al., 2009.
